# Apically extruded debris associated with ProTaper Next, ProTaper Gold and TruNatomy systems: An in vitro study

**DOI:** 10.34172/joddd.2021.006

**Published:** 2021-02-13

**Authors:** Neslihan Yılmaz Çırakoglu, Yağız Özbay

**Affiliations:** Department of Endodontics, Faculty of Dentistry, Karabük University, Karabük, Turkey

**Keywords:** Apical extrusion, ProTaper Gold, ProTaper Next, Root canal preparation, TruNatomy

## Abstract

** Background.** This research aimed to investigate and compare the amount of apically extruded debris after root canal preparation using ProTaper Next, ProTaper Gold, and TruNatomy systems.

**Methods.** Forty-five extracted mandibular premolar teeth with single canals with similar lengths were used. The root canals were prepared using ProTaper Next (Dentsply Maillefer, Ballaigues, Switzerland), ProTaper Gold (Dentsply Maillefer, Ballaigues, Switzerland), or TruNatomy (Dentsply Maillefer, Ballaigues, Switzerland) files. Apically extruded debris during preparation was gathered into preweighed Eppendorf tubes. Then the Eppendorf tubes were incubated at 70°C for five days. The Eppendorf tubes were weighed again to determine their final weight plus the extruded debris.

**Results.** The TRN system resulted in significantly less debris extrusion than the PTN system (*P* <0.05). There was no significant difference between the PTN and PTG groups and between the PTG and TRN groups (*P* >0.05).

**Conclusion.** All the instrumentation systems caused apical extrusion of debris. However, the TRN system resulted in significantly less debris extrusion than the other systems.

## Introduction

One of the fundamental steps of root canal therapy is chemomechanical preparation. It consists of mechanical disinfection with instruments and the use of irrigants. During these procedures, pulp tissue, organic tissue remnants, dentin chips, bacteria and their products, and/or irrigation solutions could be extruded into the periradicular tissues. A proper check of the working length might reduce the likelihood of this complication. However, even so, any extrusion of debris can activate an inflammatory process in the periapical area, leading to delayed healing, postoperative complications, such as flare-ups, or short-/long-term failure.^1^ Flare-up is characterized by swelling, pain, or both during and after endodontic treatment and this condition leads to unscheduled appointments of the patients.^2^ At present, overall instrument systems and preparation methods lead to the extrusion of debris.^3-6^ However, the amount of debris extrusion can alter association with the preparation method and the pattern of the file systems.^3,7-10^

ProTaper Next (PTN, Dentsply, Maillefer, Ballaigues, Switzerland) is a NiTi file system manufactured from M-Wire NiTi alloy to provide better ﬂexibility and cyclic fatigue resistance.^11^ PTN files have an off-center rectangular design and progressive and regressive tapers. Variations in taper minimize the connection between the file and the dentin, reducing the screw impression and undesirable taper lock.^12^ Also, the offset design maximizes the debris forced out of the root canal in comparison to a file with a centered mass and axis of rotation.^13^ The PTN instruments include five finishing (X1, X2, X3, X4, and X5) files.

ProTaper Gold (PTG) (Dentsply Maillefer, Ballaigues, Switzerland) system, which uses a traditional continuing rotation motion, has a convex triangular cross-section, variable progressive taper, and rotary action. It is claimed that PTG is manufactured with professional metallurgy. It has a continuously tapered shape for more efficient and safer cutting action along with reportedly increased elasticity and impedance to cyclic fatigue.^14^ The PTG system includes shaping (Sx, S1, and S2) and finishing (F1, F2, F3, F4, and F5) files.

TruNatomy, a heat‐treated NiTi system (TRN; Dentsply, Maillefer, Ballaigues, Switzerland), has been introduced recently. It is marketed with three different sizes, including small: #20, 0.04 taper; prime: #26, 0.04 taper; and medium: #36, 0.03 taper. The distinctive feature of the TRN files is slip shaping, which enables more space for debridement. The manufacturer has claimed that TRN files are more flexible and fatigue-resistant due to the special heat treatment with a special design. TRN instruments have an off-center parallelogram cross-section design.^15^ It has been claimed that TRN files conserve the structural dentin and tooth entirety because of the instrument geometry, regressive tapers, and the slenderized pattern in addition to the heat-treatment of the NiTi alloy.^15,16^

Apically extruded debris caused by root canal preparation with TRN instruments has not been evaluated yet. Therefore, this research evaluated the amount of apically extruded debris subsequent to the preparation of root canals with the ProTaper Next, ProTaper Gold, and TruNatomy systems. The null hypothesis was that there would be no significant difference between the groups in means of apically extruded debris.

## Methods

Forty-five freshly extracted human mandibular premolars with a single canal and similar root length were gathered. Teeth with root resorption, immature roots, calcification, previous root canal treatment, or root canal curvature >10º were not included in the study. The absence of calcification inside the root canals was verified radiographically. After soft tissue remnant and calculus removal from the root surfaces, the teeth were stored in normal saline solution until the experiment. Conventional coronal access cavities were prepared using a high-speed bur. The buccal cusp tips of each tooth were flattened to obtain a reference point. Apical patencies were controlled by inserting a #15 K-file (Dentsply Maillefer, Ballaigues, Switzerland) into each root canal until its tip was visible at the apical foramen, and the working length was measured by subtracting 1 mm from this length.

To collect the apically extruded debris, an experimental model described by Myers and Montgomery^17^ was used. The caps of Eppendorf tubes were removed, and the tubes were weighed using an analytical balance (Radwag, Radom, Poland) with an accuracy of 10-^4^ g for initial measurements. Three following weights were recorded for each tube, and the mean values were calculated. Round holes were created in the caps of Eppendorf tubes, and the teeth were inserted through this cap up to the cementoenamel junction. A 27-gauge needle was also inserted through the side of the cap to compensate for the inner and outer air pressure, and the pattern of the tooth and needle was fixed on the cap with cyanoacrylate (Pattex Super Glue; Türk Henkel, Inc., Istanbul, Turkey) glue to avoid irritants’ escape. These patterns were attached to their Eppendorf tubes. The tubes were placed in vials for stabilization during instrumentation. The vials were covered with aluminum foil to prevent the operator from seeing the root apex during instrumentation.

Forty-five specimen were randomly divided into three experimental groups (n=15).

*Group 1:* ProTaper Next files were used in the sequence X1 (#17, 0.04v) and X2 (#25, 0.06v) with an endodontic motor (X–Smart, Dentsply Maillefer, Ballaigues, Switzerland) according to the manufacturer’s instructions. During the preparation, a gentle in-and-out brushing motion was used until resistance was felt in the root canal. The file was then withdrawn from the root canal, cleaned, and inspected before re-use. These procedures were repeated until the file reached the WL with the file.

*Group 2:* ProTaper Gold Sx (#19, 0.04v), S1 (#18, 0.02 taper), S2 (#20, 0.04v), F1 file (#20, 0.07v), and F2 file (#25, 0.08v) were sequentially used with the same endodontic motor with slight in-and-out movements according to the manufacturer’s instructions.

*Group 3:* TruNatomy^TM^ files were used in sequence: orifice modifier (#20, 08v), glider (#17, 02v), small (#20, 0.04v), and prime (#26, 0.04v) with the same endodontic motor. The files were used with 2‒3 small amplitudes approximately 2‒5 mm in and out of the root canal. Upon reaching the working length, the files were removed to avoid over-enlargement of the apical foramen.

All the root canal preparations were conducted by the same operator to eliminate any bias. During the instrumentation procedure, each sample was irrigated with a total volume of 10 mL of distilled water between files.

The debris extrusion was assessed by another researcher who was blinded to group assignment. After completion of the root canal instrumentation, the Eppendorf tube caps were removed along with teeth. To include the debris that had adhered to the apical portion of the roots, the apices of the teeth were irrigated with 1 mL of distilled water. After storing the Eppendorf tubes in an incubator at 70°C for five days to evaporate distilled water, the tubes were weighed three times with the same method and device, and the mean values were calculated. The net weights of extruded debris were calculated by subtracting the weight of the empty Eppendorf tube calculated before from the final weights of dried tubes.

The statistical analysis of the amount of extruded debris was performed using Minitab 17 statistical software (Statistical Software Release, version 17.3.1. Minitab Inc. USA). The data were reported as mean values. The groups were compared using the Kruskal-Wallis test. The amount of apically extruded debris was analyzed statistically using ANOVA, followed by post hoc Tukey tests for multiple comparisons. *P* values<0.05 were used to indicate statistical significance for all the tests.

## Results

The amount of the extruded debris was recorded in all the groups. The mean values and standard deviations of each experimental group are presented in Table 1. The mean values for the amount of extruded debris in descending order were: group 1 (PTN) (0.0016 g), group 2 (PTG) (0.00138 g), and group 3 (TRN) (0.00116 g).

**Table 1:** The means and standard deviations (SD) of apically extruded debris in grams in each system

	**Debris Extrusion (g)**	
	**Mean**	**SD**
ProTaper Next	0.0016^a^	0.000530
ProTaper Gold	0.00138^a,b^	0.000804
TruNatomy	0.00116^b^	0.000620

Different letters indicate statistically significant difference (*P*< 0.05).

**Figure 1:**
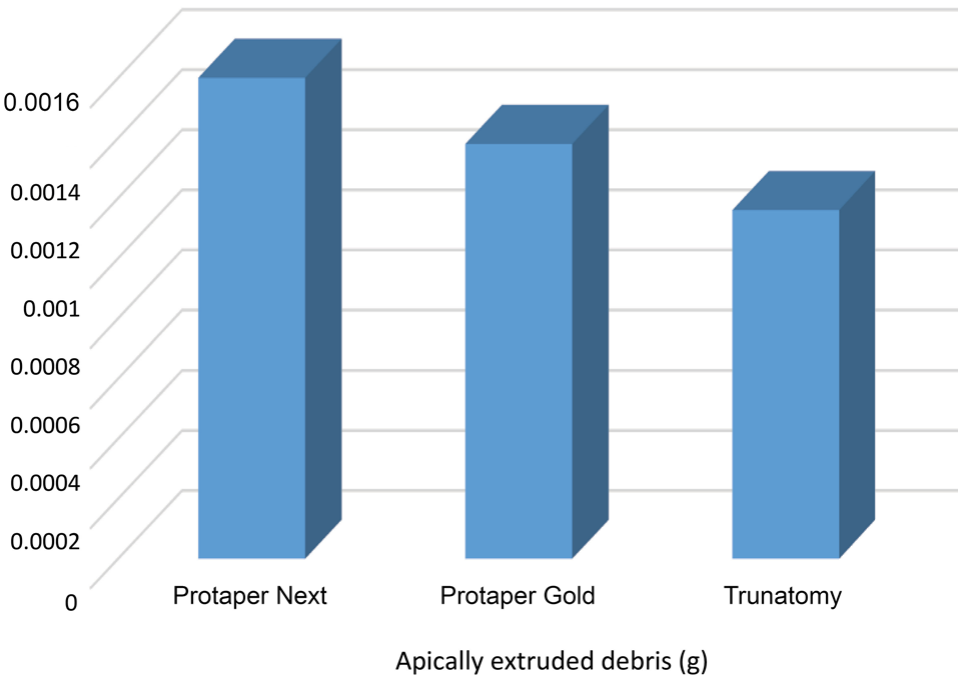
The amount of apically extruded debris after canal preparation with different instrumentation systems.

The TRN group exhibited significantly less debris extrusion than the PTN group (*P* = 0,0079, *P*< 0.05). There was no significant difference between the PTN and PTG groups and between the PTG and TRN groups (*P*> 0.05) (Figure 1).

## Discussion

During or after root canal treatment, some clinical complications, such as pain, inflammation, and slow periapical healing, are associated with apically extruded debris due to inﬂammatory reactions triggered by forcing contaminated and non-contaminated dentin and pulp tissue into periapical areas.^18^ Therefore, avoiding debris extrusion might be necessary to minimize postoperative complications. The apical extrusion of debris during chemomechanical preparation has been reported in the literature; however, many parameters affect the amount of debris extruded, such as the kinematics, preparation technique, and the design, size, and number of the instruments used in each system. However, bacterial content and antigenic characteristics of extruded material might be more crucial than the total amount of material extruded in terms of initiation of periapical response.^5^ Therefore, further studies are needed to focus specifically on shaping methods and bacterial load within the root canals.

Premolar teeth with a single root and straight canals were used to avoid working length loss or nonstandard preparation and irrigation in the curved root canals in the present study. The widely used study design of Myers and Montgomery was applied to collect apically extruded debris.^17^ The fundamental limitation of this process was that the normal periapical resistance in a tooth in clinical conditions was not simulated. This environment could be imitated using materials such as agar gel and ﬂoral foam. However, this approach has several drawbacks, including the ingress of foam and the complication in determining a certain thickness for the agar gel at the apex for mimicking the dimension of the periapical lesion.^19,20^

Some studies have reported different study methods to assess debris extrusion. Ruiz-Hubard et al^18^ used the ﬁlter column suction system and acrylic models to standardize the samples regarding curvature, shape, and size. However, acrylic models lack the components of the true nature of the root canal system, such as pulpal and canal abnormalities, three-dimensional curvatures, and an innate apical constriction. In addition, the heat generated during the instrumentation might mollify the models simulating root canals, negatively affecting the outcomes.^21^ Since sodium hypochlorite could replace the amount of extruded dentin debris by crystallization, distilled water was used as the irrigation solution^5^; 20 mL of distilled water was used during the preparation in the whole canals.

The comparison of the amount of apically extruded debris has been one of the main focuses of endodontics due to the factors mentioned above. It was found that the PTN system, another well-studied rotary system, extruded significantly less debris than the PTU system,^22,23^ which was attributed to the design of the PTN files, which ensure snake-like and swaggering motion and fewer files in the system.

Currently, there are not many studies on the PTG and PTN systems regarding apical debris extrusion. Çakıcı et al^23^ found that the PTG system extruded significantly more debris than the PTN system. In the present study, the PTN system extruded quantitatively more debris than the PTG system, without any significant difference from the study by Çakıcı et al.^23^ However, curved mesial roots were used in that study and single and straight root canals were used in the present study, which might have led to different results.

TRN system,arecently introduced nickel-titanium system, has been reportedtohave improved the performance with increased adaptation to the true nature of the tooth anatomy, preserving structural dentin and tooth integrity compared to PTN due to its slim design and regressive tapers. The manufacturer claims that the three shaping instruments, i.e., small, prime, and medium, provide a slim shaping, which improves the debridement due to more available space.^16^

The characteristics of instrument systems, such as kinematic, cross-section concept, shaping capacity, tip diameter and taper, affect the amount of apically extruded debris.^24^ Apart from the file design, several studies concluded that the number of files used during the preparation could be a factor responsible for a greater amount of extruded debris.^25,26^ In the present study, there was no association between the number of files used for canal preparation and the amount of debris extruded, consistent with the results of a previous study.^27^ In the present study, the PTG system extruded quantitatively less debris than the PTN system, with no significant difference, and extruded more debris than the TRN system, with no significant difference. Nevertheless, the results of the PTG system cannot be compared with other systems due to the lack of similar studies.

Apically extruded debris during root canal preparation with the TRN system has not been evaluated. Therefore, a direct comparison is not possible. In this study, the TRN system extruded less debris (0.00116 g) compared to the PTG (0.00138 g) and PTN (0.0016 g) systems. Also, the TRN system exhibited significantly less debris extrusion than the PTN system. Therefore, the null hypothesis was rejected. This finding might confirm the ability of the TRN system to be used in root canals with minimal access cavities and preserve the dentin tissue by a conservative technique. The results of this study are consistent with other studies since apical extrusion of debris is inevitable during the mechanical preparation of root canals.^22,28-30^ Therefore, all the instrumentation systems used resulted in apically extruded debris.

## Conclusion

Under the experimental conditions of the present study, it can be concluded that all the instrumentation systems led to apical debris extrusion. However, the TRN system resulted in significantly less debris extrusion than the PTN system.

## Authors’ Contributions

NYÇ and YÖ were responsible for the design, experiment, and data analysis, and drafting and proofreading of the manuscript.

## Funding

Not applicable.

## Competing Interests

The authors declare no competing interests with regards to the authorship and/or publication of this article.

## Ethics Approval

The study protocol was approved by the Ethics Committee of Non-Interventional Clinical Research of Karabük University, Turkey (2020/204).
